# Empathy and Coping in Allied Health Sciences: Gender Patterns

**DOI:** 10.3390/healthcare9050497

**Published:** 2021-04-22

**Authors:** Artemisa R. Dores, Helena Martins, Ana C. Reis, Irene P. Carvalho

**Affiliations:** 1 Center for Rehabilitation Research, School of Health, Polytechnic Institute of Porto, 4200-072 Porto, Portugal; 2 Departamento HM, School of Business and Economics, Lusófona University, 1749-024 Lisboa, Portugal; helenagmartins@gmail.com; 3 CEOS.PP—Porto Accounting and Business School, Polytechnic Institute of Porto, 4465-004 São Mamede de Infesta, Portugal; 4 Santa Maria Health School, 4049-024 Porto, Portugal; ana.reis@santamariasaude.pt; 5 Clinical Neurosciences and Mental Health Department and CINTESIS, Faculty of Medicine, University of Porto, 4200-319 Porto, Portugal; irenec@med.up.pt

**Keywords:** empathy, coping, gender, allied health sciences, undergraduate students

## Abstract

This study aimed to examine the patterns of associations between empathy and coping among undergraduate men and women studying at Allied Health Sciences. This cross-sectional study is part of a larger longitudinal study conducted in an Allied Health Sciences School. Participants were 183 undergraduate students from 12 training programs (e.g., Physiotherapy, Occupational Therapy, Speech Therapy). Their mean age was 20.79 years (*SD* = 2.64), and they were in their first, third, and fourth years of school. The instruments were the Brief-COPE and the Interpersonal Reactivity Index (IRI). Empathy correlated with coping strategies in both genders, though showing different patterns of association. First, distinct coping strategies were associated with the same empathy dimension (perspective taking) among women (positive reframing and self-blame) and among men (active coping). Second, the same three coping strategies appeared in both genders (seeking emotional or instrumental support and resorting to religion) but associated with different empathy dimensions (cognitive empathy among women and mostly emotional empathy among men). Third, among women (but not among men), two coping strategies (positive reframing and behavioral disengagement) were each simultaneously correlated with cognitive and emotional empathy in opposite directions. Fourth, emotional empathy correlated, only among women, with several coping strategies considered to be maladaptive (behavioral disengagement, denial and substance use). Among men, only one significant coping strategy was considered to be maladaptive (behavioral disengagement) and it was negatively correlated with cognitive empathy (perspective taking). Unlike in women, relationships between the empathic dimension of fantasy and coping strategies were non-significant among men. These distinct patterns of associations emerged despite significant differences in empathy by gender (fantasy, personal distress and empathic concern) and in coping strategies (instrumental support, emotional support, religion and venting). These results support the idea that the display of empathy might be associated with gender differences in the underlying empathy dimensions and in the coping strategies used to deal with stress in the undergraduate programs of Allied Health Sciences.

## 1. Introduction

Empathy is considered to be an essential part of the relationship with patients in healthcare professions [1]. In the field of Allied Health Sciences such as Physiotherapy, Anatomical Pathology, Audiology, Cardiopneumology, Environmental Health, Neurophysiology, Occupational Therapy, Radiology, Radiotherapy, or Speech Therapy, emotionally charged situations related with patients’ concerns and suffering are frequent. Empathy involves reacting emotionally to the patient’s emotions and understanding the patient’s experiences [1,2]. An emotional and a cognitive component are thus involved in empathy. The emotional component refers to a visceral, quick and involuntary reaction to the experiences of others. The cognitive component refers to the ability to understand the perspective of the other [2]. This sense of shared emotional state and of being understood has been shown to decrease patients’ anxiety, lead to increased satisfaction and adherence to medical recommendations, and has been associated with improved health outcomes [3,4]. However, the empathic capacity can be compromised in stressful situations, especially if students in these health professions lack the coping mechanisms that allow them to effectively deal with stress.

Research suggests that stress affects empathy, enhancing [5] or decreasing emotional empathy [6]. In situations of laboratory-induced social stress, studies report sex differences in empathic accuracy [7] or in social cognitive competence [8] (i.e., the ability to accurately infer the mental state of others), with increased accuracy and competence among stressed men, but not among women, whose accuracy decreased under stress [7]. The opposite was found in a study using the same laboratory-induced socially stressful situation, with women showing increased emotional and cognitive empathy under stress, whereas men showed decreased empathy [9]. Even though stress affects the empathic capacity, the evidence of its influence in increasing or decreasing empathy is mixed, which might be related with the coping mechanisms that men and women use. This possibility finds support in research on the neural mechanisms underlying stress and empathy.

Recent studies show that stress modulates the neural mechanisms of empathy [10,11]. Gonzalez-Liencres, Breidenstein, Wolf, and Brüne [10] posited that men and women might rely on distinct mechanisms to cope with stress. This was based on their findings that men and women presented equivalent behaviors (in empathy and in the ratings of unpleasantness attributed to a painful situation) after exposure to the same laboratory-induced socially stressful situation as above. However, they showed sex differences in the neural responses to empathy for pain [10]. Specifically, a positive correlation was found between cortisol change and differential event-related potential (ERP) components among stressed men, but not among women, implying that stress increases empathy in men. Different coping strategies might thus underlie the equivalent of empathy among men and women in the studies that report non-significant gender differences among medical students regarding empathy [12,13].

Lazarus and Folkman [14] defined coping as the individual’s constantly changing cognitive and behavioral efforts to manage external and/or internal demands in response to stress. In the literature, coping strategies are sometimes classified as adaptive and maladaptive, namely in function of their associated outcomes (e.g., levels of distress, or disease progression [15]). Adaptive coping strategies include active coping, planning, positive reframing, acceptance, humor, religion, and using emotional and instrumental support, whereas maladaptive strategies include self-distraction, denial, venting, substance use, behavioral disengagement, and self-blame [16]. However, this classification of coping mechanisms as adaptive or maladaptive might not be always clear cut. Authors have referred to, for example, the venting of emotions and behavioral and mental disengagement as “arguably” less useful coping responses [17] and a systematic literature review of factor analyses showed that religious coping was sometimes loaded together with maladaptive, and sometimes with adaptive coping strategies [18].

Previous research has shown that men and women in general tend to use different coping mechanisms when dealing with stressors. In a meta-analysis focusing on gender differences in the face of a variety of stressors (e.g., dealing with infertility, work demands, family stressors, pain, caring for a chronically ill spouse, educational transitions in academia), the authors found that women engaged in more of the 17 types of strategies identified in the literature than men did. No significant gender differences were found only for planning, denial, isolation, venting (i.e., acting out, including crying, yelling, breaking things, or using drugs), self-blame, and doing exercise, most of which are considered as maladaptive behaviors. Otherwise, women were more likely than men to focus on the problem and actively cope with the stressor, seek instrumental and emotional support, use positive reappraisal, positive self-talk and wishful thinking, and resort to religion. They were also more likely than men to both ruminate about and avoid the problem through self-distraction (e.g., doing or thinking of other things) [19].

In the areas of health, the academic demands can constitute stressful situations, and students frequently display poorer mental health than the general population and their peers who study other fields [20,21]. However, the demands of academic life and of clinical internships can have a greater or a lesser impact on students’ health and empathic capacity, also depending on the coping mechanisms used to deal with these stressors. For example, a study on burnout and coping among internal Medicine trainees reported that women used the adaptive coping mechanisms of emotional and instrumental support. However, they also used the maladaptive coping mechanism of self-blame more frequently than men [22]. The authors concluded that self-blame might explain the higher burnout levels found among women in their study.

These results suggest that men and women in the areas of health might differ from men and women in the general population regarding the coping mechanisms that they use. Little is known about the coping mechanisms that students use in the context of Allied Health Sciences. Even less is known about the relationship between empathy and coping strategies among these students. The existing research mostly focuses on students in other health areas, such as Medicine or Nursing. However, like in Medicine and in Nursing, students in Allied Health Sciences also undergo school programs that include academic and practice elements, and will have to deal with patients and their specific situations, concerns and emotions. The aim of this study was to examine the associations between empathy and coping among the undergraduate students of Allied Health Sciences, considering gender patterns. Based on the literature, we expected that the coping strategies associated with empathy would be different among men and among women.

## 2. Materials and Methods

### 2.1. Participants

Participants were 183 undergraduate students from 12 training programs (Anatomical Pathology, Audiology, Cardiopneumology, Clinical Analysis, Environmental Health, Physiotherapy, Neurophysiology, Occupational Therapy, Pharmacy, Radiology, Radiotherapy, and Speech Therapy). Their mean age was 20.79 years (*SD* = 2.64), and most were women (*n* = 141, or 77%). They were attending the first school year (73, or 38.6%), the third year (56, or 29.6%) and the fourth year (54, or 28.6%).

### 2.2. Instruments

Students completed the Brief-COPE Inventory [23] and the Interpersonal Reactivity Index (IRI) [2,24]. Socio-demographic data on all students were also obtained.

The Brief-COPE Inventory [23] is a self-report instrument designed to evaluate different coping responses. This comprises 28 items measured on a 4-point Likert scale (1—I haven’t been doing this at all; 4—I’ve been doing this a lot). It is composed of 14 subscales with two items each. The Brief-COPE Inventory has been translated and adapted to Portuguese, showing adequate psychometric properties (Cronbach *alphas* of 0.65 for active coping, 0.55 for acceptance, 0.70 for planning, 0.84 for venting, 0.81 for instrumental support, 0.72 for denial, 0.79 for emotional support, 0.67 for self-distraction, 0.80 for religion, 0.78 for behavioral disengagement, 0.74 for positive reframing, 0.81 for substance use, 0.62 for self-blame, and 0.83 for humor) [25]. In the Portuguese version, *alpha* values below 0.60 occurred only for one subscale (in the original Brief-COPE version, they occurred for three subscales), which is acceptable nevertheless, considering that the subscales comprise only two items each [25]. However, in our sample, planning showed an exceptionally low *alpha* value (*α* = 0.38). As a consequence, this subscale was not considered in the analysis, and thus 13 subscales remained.

The Interpersonal Reactivity Index (IRI) [2,24] is a self-report instrument consisting of 28 items responded on a 5-point Likert scale (from 0 to 4, indicating an increasing level of empathy). The Portuguese version comprised 24 items after a confirmatory factor analysis and displays good psychometric properties [26]. The IRI has four subscales: perspective taking (PT), referring to the tendency to spontaneously adopt the psychological point of view of others; empathic concern (EC), referring to the tendency to experience “other-oriented” feelings of sympathy and concern for the unfortunate situations of others; personal distress (PD), referring to “self-oriented” feelings of personal anxiety and unease in tense interpersonal settings; and fantasy (FT), referring to the tendency of imaginatively transposing oneself into the feelings and actions of fictitious characters in books, movies, and plays. Cronbach *alphas* obtained for each subscale in the Portuguese version were, respectively, 0.73, 0.76, 0.80 and 0.84 [26].

### 2.3. Procedure

#### 2.3.1. Data Collection

This cross-sectional study was part of a larger longitudinal study. Students were invited to participate after receiving information about the study’s goals and signed an informed consent. The data were collected after classes. Participation was voluntary, and data anonymity and confidentiality were ensured. The study was approved by the local research ethics committee of the Health School and complies with the Declaration of Helsinki.

#### 2.3.2. Data Analysis

Differences in empathy and in coping strategies by gender were inspected with descriptive statistics and *t*-tests. An additional ANOVA was conducted for an idea of how empathy and coping behaved across academic years. Pearson correlations were used to examine the relation between empathy and coping for each gender. Results are significant for *p* < 0.05. Analyses were conducted in SPSS, version 27.

## 3. Results

### 3.1. Differences in Empathy and Coping

The results show some gender differences for both empathy (Table 1) and coping (Table 2). Women scored significantly higher than men in three (out of four) empathy dimensions (empathic concern, personal distress, and fantasy) (Table 1) and only in four (out of 13) coping strategies (instrumental and emotional support, venting and religion) (Table 2).

An additional one-way ANOVA further showed that three of those four significant coping strategies (instrumental support, emotional support and venting) and the two emotional empathy dimensions (empathic concern and personal distress) registered significantly higher scores among students in the fourth academic year, compared with students in their third or first year. Another coping strategy that registered higher levels among students in the fourth other than in the other academic years was self-distraction. However, gender differences were non-significant for this latter coping strategy. The values registered for the significantly different coping strategies by year were as follows: instrumental support (*M* = 3.18; *SD* = 0.73 in the fourth year; *M* = 3.04; *SD* = 0.65 in the third year; *M* = 2.80; *SD* = 0.65 in the first year; *F*(2, 180) = 4.362; *p* = 0.014), emotional support (*M* = 3.19; *SD* = 0.74 in the fourth year; *M* = 2.85; *SD* = 0.85 in the third year; *M* = 2.77; *SD* = 0.81 in the first year; *F*(2, 180) = 4.391; *p* = 0.014), venting (*M* = 2.97; *SD* =0 0.74 in the fourth year; *M* = 2.67; *SD* = 0.69 in the third year; *M* = 2.49; *SD* = 0.68 in the first year; *F*(2, 180) = 4.391; *p* < 0.001) and self-distraction (*M* = 3.12; *SD* = 0.73 in the fourth year; *M* = 2.78; *SD* = 0.71 in the third year; *M* = 2.76; *SD* = 0.77 in the first year; *F*(2, 180) = 4.319; *p* = 0.015). The significantly different values registered for the two emotional empathy dimensions by year were as follows: empathic concern (*M* = 4.23; *SD* = 0.47 in the fourth year; *M* = 4.04; *SD* = 0.63 in the third year; *M* = 3.92; *SD* = 0.59 in the first year; *F*(2, 180) = 4.538; *p* = 0.012) and personal distress (*M* = 2.85; *SD* = 0.68 in the fourth year; *M* = 2.87; *SD* = 0.65 in the third year; *M* = 2.67; *SD* = 0.69 in the first year; *F*(2, 180) = 4.288; *p* = 0.015).

### 3.2. Associations between Empathy and Coping

Although the coefficients were small to moderate (*r*s between 0.17 and 0.35), empathy was significantly correlated with several coping strategies in both genders (Table 3). Significant associations between empathy and four coping strategies were common to the two genders. Specifically, seeking emotional and instrumental support, and resorting to religion were associated with more empathic capacity regardless of gender, albeit with different empathic dimensions. Among women, these three strategies were associated with cognitive empathy (mostly pertaining to perspective taking). Among men, they were mostly associated with emotional empathy (especially pertaining to empathic concern), although instrumental support was also correlated with the cognitive empathy dimension of perspective taking, like among women. Behavioral disengagement was the fourth common strategy and was associated with less cognitive empathy (perspective taking) in both genders, but also with emotional empathy (empathic concern) only among women. In general, the number of significant associations between coping strategies and empathy was smaller among men (seven significant correlations) than among women (13 significant correlations).

Unlike in women, relationships between the cognitive empathic dimension of fantasy and coping strategies were non-significant among men. Among women, fantasy was significantly correlated with seeking emotional support and venting. In addition, among women, cognitive empathy (perspective taking) was correlated with positive reframing, but also with self-blaming, whereas active coping was significantly related with empathy among men (Perspective Taking), though not among women. The remaining correlations pertained to emotional empathy and were significant only among women. Specifically, substance use was correlated with empathic concern and denial was associated with personal distress. Finally, two of the coping strategies mentioned above were simultaneously related to cognitive and emotional empathy in opposite directions among women, but not among men. These were positive reframing (positively associated with perspective taking and negatively with personal distress) and behavioral disengagement (associated negatively with perspective taking and positively with personal distress).

## 4. Discussion

In this study, we examined how empathy correlates with the strategies used by students in the Allied Health Sciences to cope with stress, considering the hypothesis that different correlation patterns might emerge for men and for women. Empathy is a crucial aspect of patient care [1,24], but can be compromised in stressful situations [8,9], especially if inadequate coping mechanisms are used under stress. Even though research exists on empathy and coping in areas such as Medicine or Nursing, little is known about these aspects in Allied Health Sciences. Knowledge of the associations for each gender can help cast light onto the empathic capacity of students in the undergraduate years of the Allied Health Sciences programs.

Our results indicate that empathy was correlated with coping strategies in both genders, although different association patterns emerged among men and among women, as expected. Research indicates that, compared with men, women tend to use more than 17 types of coping strategies identified in the literature [19]. Among women, in the current study, more coping strategies (ten out of the Brief-COPE’s 13) were also significantly associated with empathy than among men (five out of the Brief-COPE’s 13). These coping strategies appeared to be associated with all four empathy dimensions among women, whereas the fewer significant coping strategies among men appeared to be associated with only three empathy dimensions (no coping strategy was significantly associated with the empathic dimension of fantasy among men). In general, strategies to deal with stressful situations were associated with emotional empathy among men (except active coping and behavioral disengagement, which were solely associated with cognitive empathy). Among women, a more complex pattern of association emerged, including: (i) the same coping strategies observed among men but solely associated with cognitive empathy instead; (ii) emotional empathy associated with coping strategies that are considered less adequate; (iii) the presence of simultaneous, opposite-sign correlations between the same coping strategy and different empathy dimensions (which were absent among men); and (iv) the presence of several strategies exclusively among women (active coping was the only strategy appearing exclusively among men).

Regarding the specific association patterns, the same empathic dimensions in both genders were associated with the use of different coping strategies by men and by women in situations of stress. For example, the cognitive empathy dimension of perspective taking registered non-significant gender differences in our study. However, it was (positively) correlated, in the respective gender, with three coping strategies that did not emerge in the other gender, namely seeking the positive aspects of a stressful situation (positive reframing) and self-blaming among women, and taking action to deal with the situation (active coping) among men. This supports the idea, suggested in previous research [10], that men and women might resort to different coping strategies under stress, even though the behavioral result (e.g., the display of empathy) and the underlying empathy dimension (e.g., cognitive empathy) might be the same in both genders.

Conversely, when greater empathic capacity in both genders was associated with the use of the same strategies to deal with stress by men and by women, the empathy dimensions involved were different. Specifically, when instrumental support, emotional support, or religion were used (by both men and women in our study), emotional empathy (empathic concern and personal distress) was significantly higher among men, whereas solely cognitive empathy (perspective taking and fantasy) was significantly higher among women. In contrast, behavioral disengagement was associated solely with cognitive empathy (perspective taking) among men but with both cognitive and emotional empathy among women. Thus, the same coping strategies can be associated with different empathy dimensions in each gender, even though the behavioral result (e.g., the display of empathy) might appear to be the same in men and in women.

The additional four coping strategies that were significantly associated with empathy levels among women (though not among men) were mainly related with women’s emotional empathy. The only significant (positive) association exclusively observed with a cognitive dimension of empathy in this set referred to the tendency to imagine oneself in fictional situations (fantasy). The more women used the venting of emotions, the higher their fantasy levels, which is an empathy dimension that is not considered as essential in patient care [12,27]. Otherwise, the greater the women’s personal distress, the more they used denial to deal with stressful situations, and the less they used humor. The greater women’s empathic concern, the more they resorted to substance use in situations of stress. This means that strategies to deal with stress that are considered as maladaptive (e.g., denial, behavioral disengagement, mentioned above, and substance use) [16] were associated with greater emotional empathy among women (but not among men). Among men, the only coping strategy which was considered to be maladaptive was behavioral disengagement, and it was associated with cognitive, not with emotional, empathy (the greater the men’s disengagement, the lesser their capacity for perspective taking).

This same coping strategy (behavioral disengagement) showed opposite associations, respectively, with women’s emotional and cognitive empathy. The more women used behavioral disengagement in situations of stress, the higher their levels of emotional empathy (empathic concern) and the lower their levels of cognitive empathy (perspective taking). This underscores the relative independence of the four empathy dimensions and their manifold possible constellations [2], although this result concerns the two prototypical dimensions of empathy [27] that are directed at others [12] and considered as essential in healthcare settings. The strategy of positive reframing, discussed earlier, was also simultaneously associated with cognitive and emotional empathy in opposite directions. The greater women’s use of this strategy, the higher their levels of Perspective Taking and the lower their levels of personal distress, reflecting the divide that emotional resonance can produce, by triggering sympathetic pain or, alternatively, initiating altruism and compassion [28]. It has been suggested that professional empathy involves a shift from feeling with the patient’s suffering to a curiosity about the patient’s circumstances [28]. Positive reframing in situations of stress might be used to that effect. No such simultaneous opposite associations were observed among men.

Empathy and coping were correlated both among men and among women, despite the differences observed between the genders when empathy and coping were each considered separately. In our sample, the women scored significantly higher than men in empathy, which is consistent with previous studies [2,12,27]. The exception was perspective taking, which registered non-significant gender differences, like in research reporting no gender differences among undergraduate students [29] and medical students [13,30]. The higher levels of emotional empathy observed among students in their fourth academic year, compared with students in their third and first years, are at odds with longitudinal research reporting declines in empathy after the third year among medical students [31,32]. In the future, the longitudinal project of which this study is a part will make it possible to further inspect this difference regarding empathy throughout school years and also by gender. Regarding coping strategies, except for venting (which women in our study used more frequently than men did), the gender differences in our sample followed the pattern reported in the general population [19] more closely than that observed specifically among internal medicine trainees, namely regarding religion [22]. It is possible that the difference in venting from the general population has to do with the comparatively more restricted nature of this concept in the Brief-COPE Inventory used in our study. For example, in Tamres, Janicki, and Helgeson’s study [19], the category of venting was broader and included substance use. Longitudinal research suggests that, from admissions to the end of their third year, medical students had shifted to using active strategies less (including seeking social support) and relying on emotional strategies more [33]. The differences by year in the current study align with the latter finding (namely regarding venting), though not with the former (namely regarding instrumental and emotional support). This difference between medical and Allied Health Sciences students merits further attention, although a longitudinal approach will allow more adequate comparisons between studies, as mentioned above.

In sum, among the students in Allied Health Sciences, active coping and positive reframing in situations of stress were each associated with greater cognitive empathy (perspective taking), respectively, among men and women. In addition, using instrumental support, emotional support or religion in stressful situations was associated with greater emotional empathy among men, and exclusively with cognitive empathy among women. Unlike among men, emotional empathy (including empathic concern) among women was positively associated with strategies to deal with stress that are considered as maladaptive (including behavioral disengagement, denial and substance use). Two additional strategies considered to be maladaptive were associated with cognitive empathy among women as well (self-blame and venting). Additionally, among women (but not among men), some strategies used to deal with stress were simultaneously associated, but in opposite directions, respectively, to cognitive and emotional empathy. This suggests that, under stress, women may simultaneously be more and less empathic, depending on the (emotional or cognitive) dimension considered, and on the coping strategy used. This “paradox” was not observed among the men in this study. Of all the significant strategies used to deal with stress among men, most were positively associated with emotional empathy, two were positively associated with cognitive empathy and only one (negatively correlated with cognitive empathy) was considered as maladaptive [16].

Further research is necessary for a better understanding of the differences observed between medical and Allied Health Sciences students. Among Allied Health Sciences students, more research is needed to explore the associations between greater empathy and the use of coping strategies that are considered maladaptive. If such coping strategies are associated with higher empathy levels, like in this study, they may be useful and might be a natural or necessary part of dealing with stress, namely for women who are emotionally empathetic. However, in the context of healthcare professions, self-blame might be associated with a greater risk of burnout [22]. Our results also suggest that using, at least, denial or behavior disengagement to deal with stress was associated with somehow hindered empathy toward the patient. Specifically, denial in situations of stress was positively correlated with personal distress, an empathy dimension that has been considered as self-referenced, rather than as patient-oriented, and that shows negative correlations with perspective taking in several studies [2,34]. Personal distress can pose a risk to the shift in focus that is necessary in healthcare professions, from feeling with the patient’s suffering to attending to the patient’s circumstances [28].

In turn, the simultaneous positive and negative associations between behavior disengagement and empathic concern and perspective, respectively. The results seem to indicate that empathy is only partially at work when this coping strategy is used. These are considered the two other-oriented dimensions of empathy [34] that are relevant in healthcare professions, fostering the emotional responsiveness that helps to build trust, as well as the understanding of the patient’s perspective [12,28].

This study provides a picture of the relation between participants’ generic empathy and their general strategies to deal with stress. Studies in which participants undergo actual, concrete stress situations, inspecting the strategies that they use to deal with that stress and associated (emotional and cognitive) empathy, could help illuminate our results (namely regarding maladaptive coping), including in terms of the outcomes of the situation at the moment and over time. The results for different academic years, in particular, are based on a cross-sectional approach, and future longitudinal studies will allow assessment throughout the years. In addition, future studies are warranted to inspect whether these results are replicable in other settings. The current study included one school and a smaller number of men than of women, which is a trend in health courses. Still, this limits the generalizability of the results and could be a potential source of bias, namely regarding the different patterns of associations observed for each gender in this study. A power analysis of this sample revealed values below 30% among the group of men for the empathy and coping dimensions that registered non-significant gender differences. Associations between empathy and coping might thus be visible with a larger sample that also includes more men. Nevertheless, the gender analysis in this study had the power to suggest that there are differences between men and women regarding the constructs of the study, which deserves further attention in future research.

## 5. Conclusions

Among students in Allied Health Sciences, men and women use the same strategies to deal with stress, however associated with different (emotional versus cognitive) empathy dimensions. In addition, men and women use different coping strategies associated with the same (cognitive) empathy dimension and also with different emotional empathy dimensions. An implication is that gender differences in empathy under stress stem not only from different strategies that men and women use to deal with stress, but also from the empathy dimensions that, in men and in women, are differently associated with the strategies used to deal with stress. Coping strategies that are considered maladaptive require further research.

## Figures and Tables

**Table 1:** Empathy by gender.

IRI Subscales	Gender	*n*	*M*	*SD*	*t*
Cognitive Empathy					
Perspective Taking	Women	141	3.81	0.53	1.83
	Men	42	3.65	0.55	
Fantasy	Women	141	3.65	0.78	3.398 *
	Men	42	3.18	0.82	
Emotional Empathy					
Personal Distress	Women	141	2.88	0.66	3.616 **
	Men	42	2.46	0.64	
Empathic Concern	Women	141	4.14	0.54	3.861 **
	Men	42	3.75	0.63	

Note. * *p* ≤ 0.05; ** *p* ≤ 0.001.

**Table 2:** Coping strategies by gender.

Brief-COPE Subscales	Gender	*n*	*M*	*SD*	*t*
Instrumental Support	Women	141	3.06	0.73	2.745 *
	Men	42	2.71	0.72	
Emotional Support	Women	141	3.10	0.75	6.154 **
	Men	42	2.30	0.73	
Venting	Women	141	2.79	0.84	3.608 **
	Men	42	2.35	0.70	
Active Coping	Women	141	3.41	0.50	1.806
	Men	42	3.25	0.52	
Positive Reframing	Women	141	2.92	0.74	−0.250
	Men	42	2.95	0.88	
Self-Blame	Women	141	2.87	0.64	0.582
	Men	42	2.80	0.73	
Acceptance	Women	141	2.85	0.69	1.398
	Men	42	2.69	0.59	
Denial	Women	141	1.89	0.73	0.488
	Men	42	1.82	0.85	
Self-Distraction	Women	141	2.89	0.78	0.606
	Men	42	2.81	0.66	
Behavioral Disengagement	Women	141	1.34	0.53	0.418
	Men	42	1.30	0.55	
Religion	Women	141	1.92	0.72	3.052 *
	Men	42	1.49	0.65	

Note. * *p* ≤ 0.05; ** *p* ≤ 0.001.

**Table 3:** Associations between empathy and coping.

	Brief-COPE Subscales												
IRI	IS	ES	Rel	AC	PR	BD	SB	Hum	Den	SU	Vent	ACC	SD
**Women** **(*n* = 141)**													
**Cognitive** **Empathy**													
**PT**	0.17 *	0.20 *	0.23 **	0.11	0.31 **	−0.30 **	0.21 *	0.07	−0.13	−0.05	0.10	0.12	0.05
*C.I.*	0.00–0.24	0.03–0.26	0.04–0.25	−0.06–0.29	0.11–0.34	−0.46–(−0.14)	0.03–0.31	−0.07–0.16	−0.22–0.03	−0.41–0.24	−0.05–0.20	−0.04–0.22	−0.08–0.15
**FT**	0.14	0.25 **	0.13	−0.05	−0.03	−0.11	0.13	0.01	0.09	−0.07	0.24 **	−0.06	−0.04
*C.I.*	−0.03–0.33	0.09–0.43	−0.03–0.28	−0.34–0.18	−0.21–0.14	−0.41–0.08	−0.05–0.36	−0.16–0.18	−0.08–0.28	−0.66–0.29	0.08–0.44	−0.26–0.13	−0.21–0.13
**Emotional** **Empathy**													
**PD**	0.05	0.06	−0.05	0.09	−0.18 *	0.16	0.01	−0.17 *	0.23 **	−0.05	−0.03	−0.16	0.12
*C.I.*	−0.11–0.20	−0.09–0.20	−0.17–0.09	−0.33–0.11	−0.31–(−0.01)	−0.00–0.41	−0.17–0.18	−0.29–(−0.01)	0.06–0.36	−0.51–0.29	−0.19–0.12	−0.31–0.01	−0.04–0.24
**EC**	0.11	0.16	0.08	0.07	0.06	0.19 *	0.09	−0.09	−0.01	0.19 *	0.08	−0.03	−0.17
*C.I.*	−0.04–0.21	−0.00–0.24	−0.06–0.16	−0.10–0.26	−0.08–0.17	−0.37–(−0.03)	−0.07–0.22	−0.18–0.05	−0.13–0.12	−0.69–(−0.04)	−0.07–0.19	−0.15–0.11	−0.23–0.01
Men **(*n* = 42)**													
**Cognitive** **Empathy**													
**PT**	0.34 *	0.18	−0.02	0.35 *	−0.03	−0.32 *	0.18	0.04	0.11	0.12	0.21	−0.01	−0.06
*C.I.*	0.03–0.49	−0.15–0.32	−0.23–0.26	0.06–0.68	−0.22–0.18	−0.62–(−0.2)	−0.10–0.37	−0.18–0.22	−0.14–0.27	−0.23–0.49	−0.09–0.44	−0.29–0.29	−0.31–0.21
**FT**	0.23	0.18	0.12	−0.28	0.01	0.18	−0.01	0.11	0.02	−0.01	0.01	−0.09	0.05
*C.I.*	−0.09–0.62	−0.15–0.56	−0.23–0.51	−0.92–0.05	−0.29–0.31	−0.21–0.73	−0.37–0.35	−0.20–0.40	−0.29–0.33	−0.56–0.54	−0.39–0.42	−0.57–0.31	−0.34–0.46
**Emotional** **Empathy**													
**PD**	−0.08	0.18	0.32 *	−0.24	−0.07	0.15	−0.20	−0.29	0.11	−0.09	−0.04	−0.06	0.14
*C.I.*	−0.36–0.21	−0.12–0.43	0.02–0.57	−0.68–0.08	−0.28–0.18	−0.19–0.54	−0.45–0.10	−0.44–0.01	−0.16–0.32	−0.55–0.30	−0.35–0.28	−0.41–0.28	−0.18–0.44
**EC**	0.35 *	0.35 *	0.34 *	0.08	0.09	−0.19	0.03	−0.08	0.14	0.09	0.13	−0.10	−0.23
*C.I.*	0.04–0.57	−0.01–0.51	0.03–0.57	−0.29–0.47	−0.17–0.29	−0.58–0.14	−0.25–0.30	−0.29–0.17	−0.13–0.34	−0.29–0.54	−0.18–0.44	−0.44–0.23	−0.51–0.08

* *p* ≤ 0.05; ** *p* ≤ 0.01; C.I. = confidence intervals; IRI: PT = perspective taking; EC = empathic concern; PD = personal distress; FT = fantasy; Brief-COPE: IS = instrumental support; ES = emotional support; Rel = religion; AC = active coping; PR = positive reframing; BD = behavioral disengagement; SB = self-blame; Hum = humor; Den = denial; SU = substance use; Vent = venting; ACC = acceptance; SD = self-distraction.

## Data Availability

On request, the first author will supply the associated data.
